# Epidemiology of tsutsugamushi disease and its relationship with meteorological factors in Xiamen city, China

**DOI:** 10.1371/journal.pntd.0008772

**Published:** 2020-10-15

**Authors:** Li Luo, Zhinan Guo, Zhao Lei, Qingqing Hu, Min Chen, Fanghua Chen, Zeyu Zhao, Jia Rui, Xingchun Liu, Yuanzhao Zhu, Yao Wang, Meng Yang, Tianmu Chen

**Affiliations:** 1 State Key Laboratory of Molecular Vaccinology and Molecular Diagnostics, School of Public Health, Xiamen University, Xiamen City, Fujian Province, People’s Republic of China; 2 Xiamen Center for Disease Control and Prevention, Xiamen city, Fujian Province, People’s Republic of China; 3 Division of Public Health, School of Medicine, University of Utah, Presidents Circle, Salt Lake City, Utah, United States of America; University of Oxford, UNITED KINGDOM

## Abstract

Tsutsugamushi disease (TD) is an acute infectious disease caused by *Orientia tsutsugamushi*. This study aimed to analyze the epidemiological features of TD, investigate chigger mites and their hosts, and investigate the meteorological factors affecting TD incidence and the host of *O*. *tsutsugamushi* in Xiamen city, China. Data on reported TD cases were collected from 2006 to 2018. Spearman’s correlation test were used for identifying the relationship between meteorological factors and TD incidence and whether meteorological factors affect the host of *O*. *tsutsugamushi*. The incidence of reported TD increased gradually from 2006, reached a peak of 4.59 per 100,000 persons in 2014, and then decreased gradually. The TD incidence was seasonal, with epidemic periods occurred mainly in summer and autumn. Patients aged 40–60 years had the highest proportion of cases, accounting for 44.44% of the total cases. Farmers had the largest number of cases among all occupational groups. *Rattus Norvegicus* was the most common host, accounting for the largest proportion of rats (73.00%), and the highest rat density was observed in March and October every year. There were significant positive correlations between the number of reported cases and average temperature, sunshine duration, and rainfall as well as between rat density and average temperature. On phylogenetic analysis, 7 sequences of hosts and human TD cases obtained from health records demonstrated the highest similarities to the Kato, Karp, and Gilliam strains. No correlations were observed between rat density, and sunshine duration and rainfall. The transmission of TD in Xiamen city, China, was seasonal, and its incidence was affected by several meteorological factors including average temperature, sunshine duration, and rainfall. However, the host of *O*. *tsutsugamushi* was only affected by average temperature.

## Author summary

Tsutsugamushi disease (TD) is a natural focal disease caused by *Orientia tsutsugamushi*, which is widespread in some areas. The incidence of TD is greatly influenced by meteorological factors. Therefore, it is essential to analyze the epidemiological features of TD, investigate chigger mites and their hosts, and explore the relationship between meteorological factors and TD incidence and whether meteorological factors affect the host of *O*. *tsutsugamushi* in Xiamen city, China. The results showed significant positive correlations between the number of reported cases and average temperature, sunshine duration, and rainfall. A positive correlation between rat density and average temperature. No correlations were observed between the rat density and sunshine duration and rainfall. We believe our findings may provide scientific basis for studies or health strategies conducted in areas that are meteorologically similar in characteristics with Xiamen city, China.

## Introduction

Tsutsugamushi disease (TD), also known as scrub typhus, is an acute infectious disease caused by *Orientia tsutsugamushi* (*Rickettsia tsutsugamushi*). It is a zoonotic disease, with rodents as the main source of infection and chigger mite larvae as the vector. People infected with TD may develop various systemic symptoms and reactions, including fever, cutaneous rash, lymphadenopathy, and elevations of C-reactive protein (CRP) and liver enzymes [1].

Globally, TD is widely distributed in southern Asia in a triangular form, from northern Japan and far eastern Russia in the north to northern Australia in the south and to Pakistan and Afghanistan in the west along with the islands of the western Pacific and the Indian Ocean [2]. More than half (55%) of the world’s population is in areas where TD is endemic, and approximately 1 billion people are estimated to be at risk for the disease [3]. In Southeast Asia, TD is a leading cause of treatable non-malarial febrile illness [4].

TD incidence was initially thought to be influenced by climate [5]. Currently, very little information is available about the relationship between meteorological factors and the incidence of TD and regarding the influence of meteorological factors on the host of *O*. *tsutsugamushi*, and the conclusions reported in published literatures remain inconsistent. For instance, in Korea, an increase in TD infection was strongly associated with the changes in meteorological conditions [6]. A study conducted in Guangzhou, China also found the seasonality of scrub typhus, suggesting that meteorological variables might affect the incidence of TD [7]. However, a study in Taiwan found a non-significant association between rainfall and TD [8]. The El Niño Southern Oscillation (ENSO) has been linked to the increased rodent populations, with higher rodent-borne diseases, such as plague and hantavirus pulmonary syndrome (HPS) [9,10]. The same effects might apply to the TD transmission. Therefore, there is an urgent need for investigating the relationship between meteorological factors and the incidence of TD and the host of *O*. *tsutsugamushi* to help with the development of an early warning system for TD.

In this study, we aimed to explore the relationship between meteorological factors and the incidence of TD and host of *O*. *tsutsugamushi*. We also estimated the effects of diverse climate variables, including average temperature, sunshine duration and rainfall, on the incidence of TD from 2006–2018 as well as on the host of *O*. *tsutsugamushi* from 2008–2017 in Xiamen city, China.

## Materials and methods

### Ethics statement

This effort of disease control was part of the Xiamen Center for Disease Control and Prevention’s routine procedure in Xiamen city, China. Therefore, institutional review and informed consent were not required for this study. All the data analyzed were anonymized.

### Study area

Xiamen city is located on the southeast coast of China, near 118°04'04" east longitude and 24°26'46" north latitude. It has a land area of 1,699.39 km^2^ and a sea area of more than 300 km^2^. Xiamen city is comprised of Siming District, Huli District, Tong’an District, Jimei District, Haicang District, and Xiang’an District, with over 4.01 million residents in 2019. The climate of Xiamen city is characterized by a subtropical monsoon climate, which is mild and rainy, with an annual average temperature of 21°C, without severe cold in the winter and severe heat in the summer. The annual average rainfall is about 1,200 mm, with the most rainfall happening from May to August every year (S1 Fig).

### Data of reported cases

We collected the reported incidence of TD data in Xiamen city from 2006–2018 by referring to the health records from the Xiamen Center for Disease Control and Prevention. We also established a dataset of the reported TD cases from 2006–2018 which came from a passively reported system. The data included gender, age, address, occupation, and the illness onset date of each case, which was collected from the Disease Prevention and Control Information System of China.

### Investigation of chigger mites and their hosts

From April to September every year between 2008 and 2018, host and vector surveys were conducted in Jimei District, Haicang District, and Tong’an District. In each district, 20 households were selected in the residential area, 5 cages (traps) were arranged indoors for each household for 3 consecutive days each month. In the outdoor area, 50 cages were arranged for 3 consecutive days each month in the mountain or farmland far away from the village houses. The rodent density was calculated by dividing the number of rodents captured by the total number of cages. All the captured rodents were recorded in detail in terms of their species, age, sex and other host biological indicators. The ears of the dead rodents were then stored in a refrigerator at 4°C, and chigger mites were collected and identified morphologically.

As there were no data on the classification of the hosts before the year 2012, we only calculated the community structure and similarity of the hosts after 2012. Moreover, as there were no available data for June, August, October, and December, 2018, we could not estimate the relationship between rat density and meteorological factors in 2018.

### Typing of *O*. *tsutsugamushi*

Genomic DNA samples were extracted from the liver tissues of host animals and human TD cases using DNA extraction kits (Tiangen, Beijing, China), followed by nested polymerase chain reaction (PCR) detection and agarose gel electrophoresis. Sequencing was conducted in fragments of around 150bp, with the primers are shown in S1 Table.

The whole blood samples of TD patients and *O*. *tsutsugamushi* DNA (OtDNA) from the liver of host animals were identified using nested PCR. Using OtDNA as a template and based on the gene fragment design of the specific surface antigen Ot56KD protein, we sequenced and analyzed the positive PCR fragments.

The phylogenetic analysis of *O*. *tsutsugamushi* genotypes was conducted based on the 7 sequences of hosts and human TD cases that were obtained in this study, along with 35 relevant reference sequences from previous studies that were obtained from GenBank, which included strains such as Karp, Kawasaki, Gilliam, Kato, and so on [11–13]. A total of 42 nucleotide sequences were aligned based on the translated amino acid sequences using ClustalW [14] implemented in MEGA v7 software [15]. Maximum Likelihood (ML) phylogenetic inference was conducted for the final alignment using RAxML v8.2.12 [16] on the CIPRES Science Gateway [17], with the best-fit model of GTR+I+G estimated by using jModelTest v2.1.6 [18] according to the Akaike information criterion. Statistical support of clades was obtained using 1000 bootstrap pseudo-replicates.

### Data of meteorological factors

Meteorological data, including daily average temperature (in degrees centigrade), sunshine (in hours of daylight) and rainfall (in millimeters) were obtained from January 2006 to December 2018 by referring to the documentation of the Xiamen Meteorological Bureau.

### Statistical methods

According to our previous study [19,20], a new method including 6 indices (including richness index *N*, Simpson diversity index *D*, Shannon diversity index *H*, Berger-Parker dominance index *d*, Shannon evenness index *E*, and Morisita-Horn similarity index *C*) were used to describe the occupational characteristics and host of TD.

The richness index *N* indicates the number of occupation classifications or host species involved in TD cases in a certain region or year. The Simpson diversity index *D* and Shannon diversity index *H* are obtained by calculating the proportions of various occupations or host [21–23]. If *D* is closer to 1 or *H* is larger, the diversity will be greater. The calculation formulas of *D* and *H* are as follows:

$$D=1-\sum_{n=1}^{N}{Pi}^{2}$$ (1)

$$H=-\sum_{n=1}^{N}Pi\;\ln\;pi$$ (2)

The Berger-Parker dominance index *d* is the proportion of the dominant occupation of TD in a certain area, and a larger *d* value indicates stronger dominant occupation or host. The calculation formula is as follows:

$$d=max(p_{i})$$ (3)

Where, *p*_*i*_ refers to the proportion of the *i*^*th*^ classification of occupation or host.

The Shannon evenness index *E* is also called Shannon’s equitability, which can be calculated using *H* and *N* [23]. The value of *E* close to 0.5 indicates better equitability. The calculation formula of *E* is as follows:

$$E=\frac{H}{\ln\;N}$$ (4)

The similarity between regions is represented by the Morisita-Horn index *C*. *C* is obtained by calculating the types of occupations or host [23]. The value of *C* close to 1 indicates greater similarity. The calculation formula is as follows:

$$C=\frac{2\sum n_{1i}n_{2i}}{(\lambda_{1}+\lambda_{2})M_{1}M_{2}},$$ (5)

$$\lambda_{i}=\frac{\sum n_{ji}^{2}}{M_{j}^{2}}$$ (6)

Where, *n*_*ji*_ represents the number cases of *i*^*th*^ occupation in the region *j*, or of *i*^*th*^ host in the year *j*. *M*_*j*_ is the total of the cases for all occupations in the region *j*, or the total of the host in the year *j*.

### Statistical analysis

Data were entered in Microsoft Excel 2013 (Microsoft Corp., USA). Data analysis was performed using SPSS 13.0 software (IBM Corp., Armonk, NY, USA). Correlations analysis between meteorological factors and reported incidence of TD cases and between meteorological factors and rat density were calculated using Spearman’s correlation test.

## Results

### Epidemiological characteristics of TD

**Fig 1:**
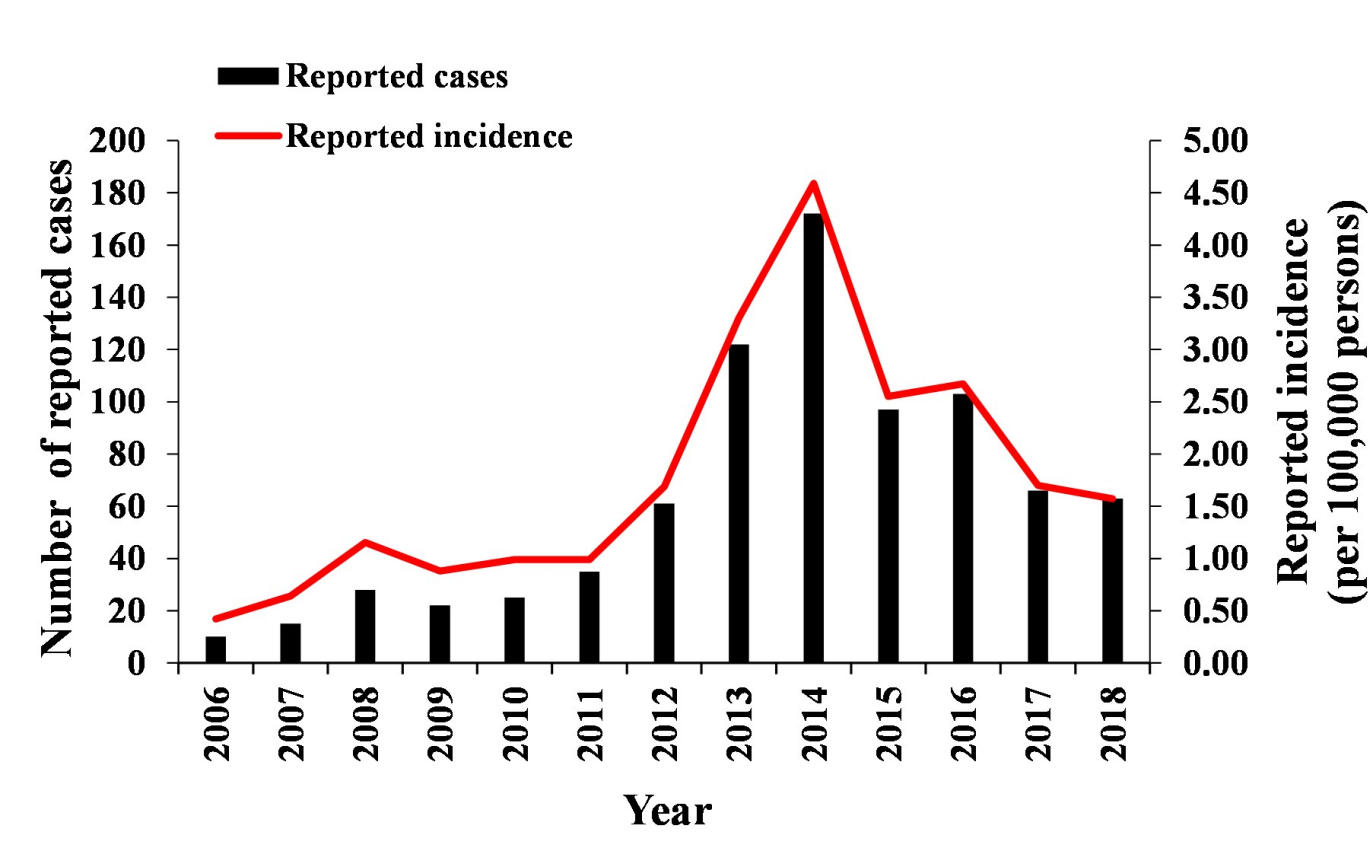
Number of reported cases and reported incidence in Xiamen city, China, 2006–2018.

We collected the reported incidence data of TD in Xiamen city from 2006–2018 by referring to the Health Records of Xiamen. From 2006 to 2018, TD incidence gradually increased since 2006, peaked at 4.59 (per 100,000 persons) in 2014 and then gradually decreased, but the incidence remained at a high level, with an overall initial trend of increasing and then decreasing (Fig 1).

**Fig 2:**
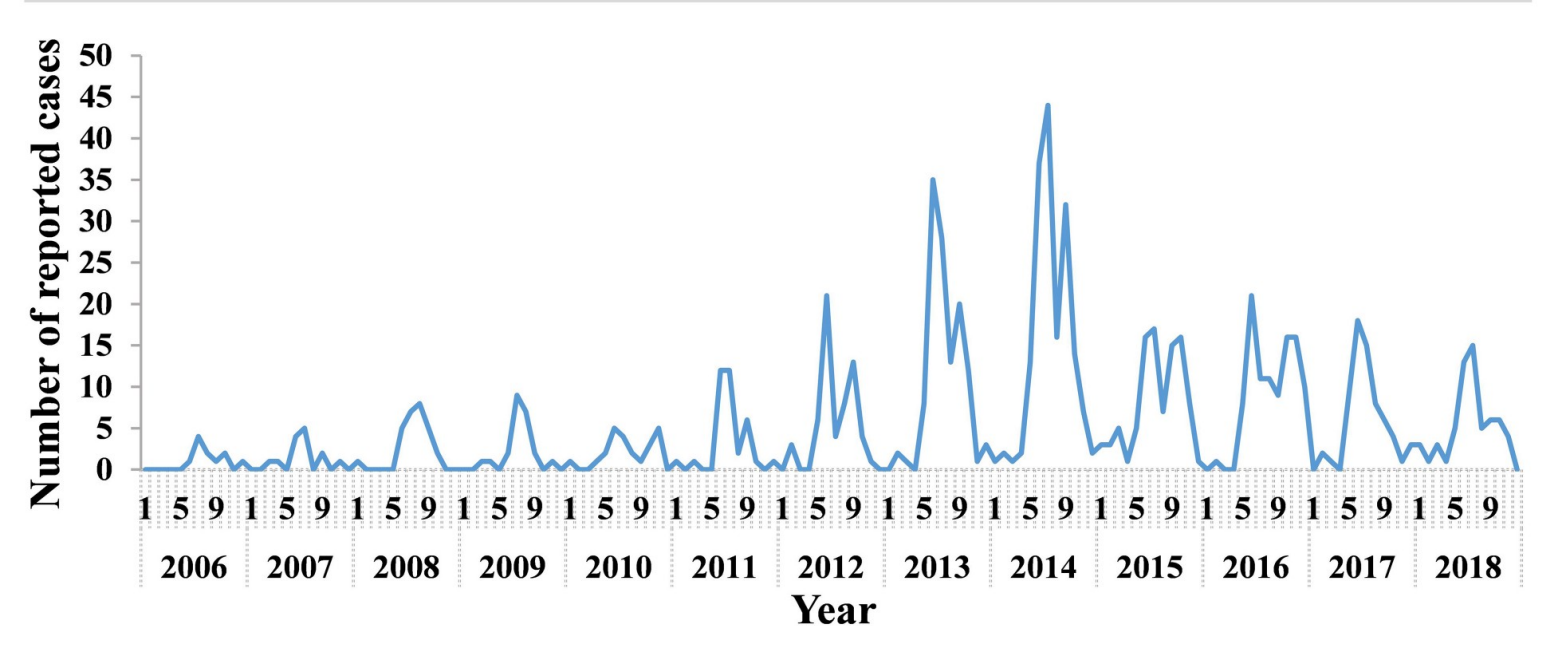
Month distribution of TD cases, Xiamen city, China, 2006–2018.

The seasonal distribution of TD is shown in Fig 2. The results showed that the epidemic periods of the TD were mainly in the summer and autumn of each year, accounting for 74.5% (610/819) of the total number of cases from May to October, with a peak epidemic in July (an average of 184 cases), accounting for 22.5% of the total number of cases.

**Fig 3:**
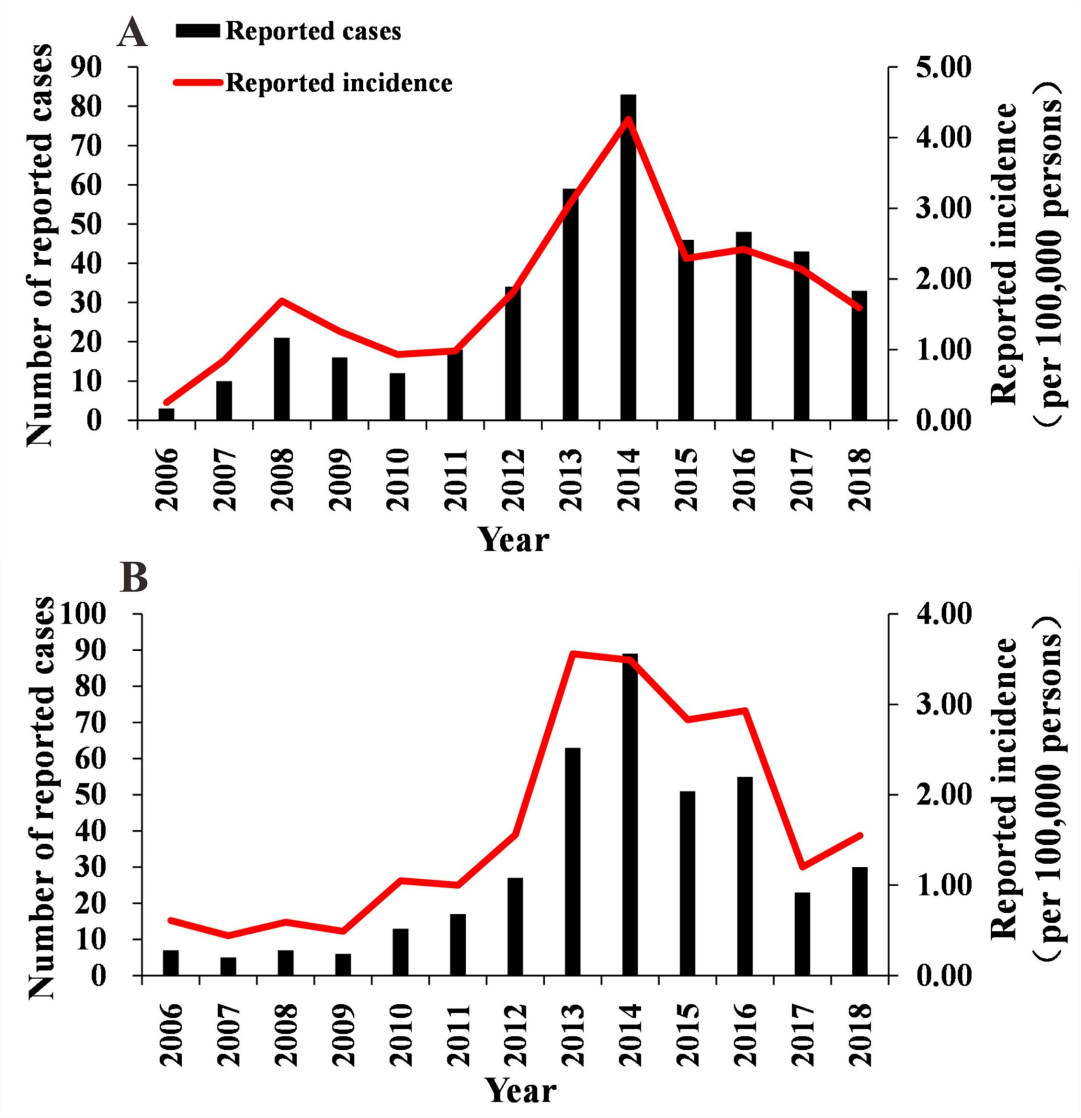
Gender distribution of TD cases, Xiamen city, China, 2006–2018. A, Men distribution of TD cases; B, Women distribution of TD cases.

**Fig 4:**
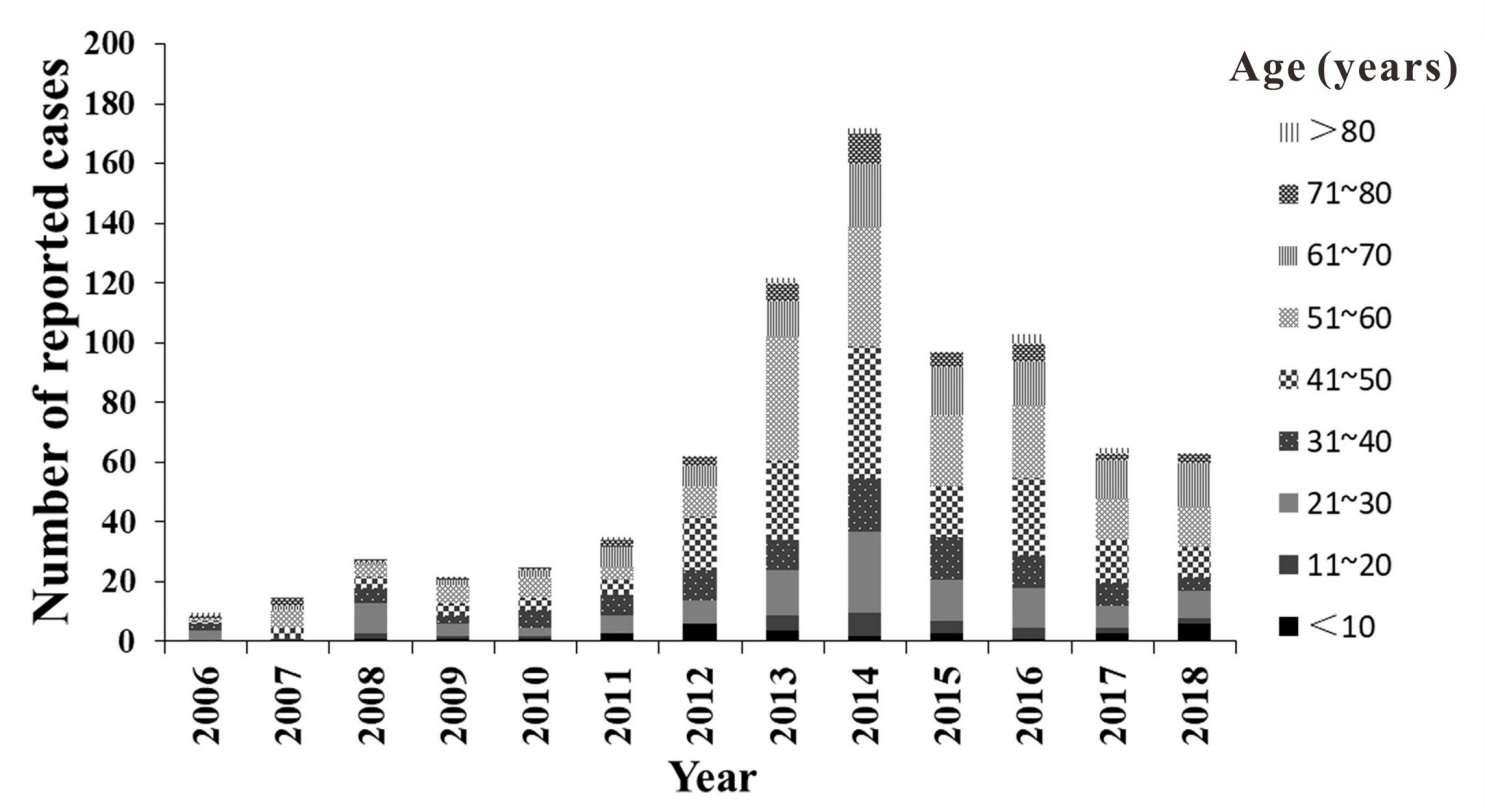
Age distribution of TD cases, Xiamen city, China, 2006–2018.

Men accounted for 52.01% (426/819) of all the cases, which was higher than the cases involving women (Table 1), but the difference was not statistically significant (*χ*^*2*^ = 0.057, *P* = 0.812). The results showed that the incidence trend of men and women was slightly different. As for men, since 2006, the incidence gradually increased, and a small peak appeared in 2008. The incidence reached a peak of 4.26 (per 100,000 persons) in 2014, after which the incidence gradually declined. As for women, the incidence of TD increased gradually since 2006, peaked at 3.56 (per 100,000 persons) in 2013, and then declined gradually. The incidence of TD in men and women both showed an overall initial trend of increasing and then decreasing (Fig 3). In our study, the age of the reported cases were 1–87 years old, among which the age group of 40–60 years accounted for the highest proportion, with 45.42% (372/819) of the total number of cases. The age group > 80 years accounted for the lowest proportion, with 1.34% (11/819) of the total number of cases (Table 1 & Fig 4). In terms of occupational categories, farmers accounted for the highest proportion of cases, at 21.00% (172/819), followed by housework and unemployment, with a proportion of 20.02% (164/819) (Table 1).

**Table 1:** Epidemiological characteristics of 819 reported TD cases, Xiamen city, China, 2006–2018.

Variables	Number of cases	Percentage (%)
Sex	819	100.00
Male	426	52.01
Female	393	47.99
Age (years)	819	100.00
<10	31	3.79
11~20	30	3.66
21~30	120	14.65
31~40	99	12.09
41~50	178	21.73
51~60	194	23.69
61~70	112	13.68
71~80	44	5.37
81~90	11	1.34
*D*_*ID*_ (days)	819	100.00
0~2	163	19.90
3~5	146	17.83
6~10	265	32.36
>10	245	29.91
Occupation	819	100.00
Unknowing	92	11.23
Catering industry	6	0.73
Cadre staff	13	1.59
Worker	81	9.89
Housework and unemployment	164	20.02
Teacher	6	0.73
Retired	28	3.42
Migrant worker	29	3.54
Farmer	172	21.00
Others	130	15.87
Diaspora children	16	1.95
Business services	34	4.15
Student	40	4.88
Childcare	7	0.85
Fishing (boat) people	1	0.12

**Fig 5:**
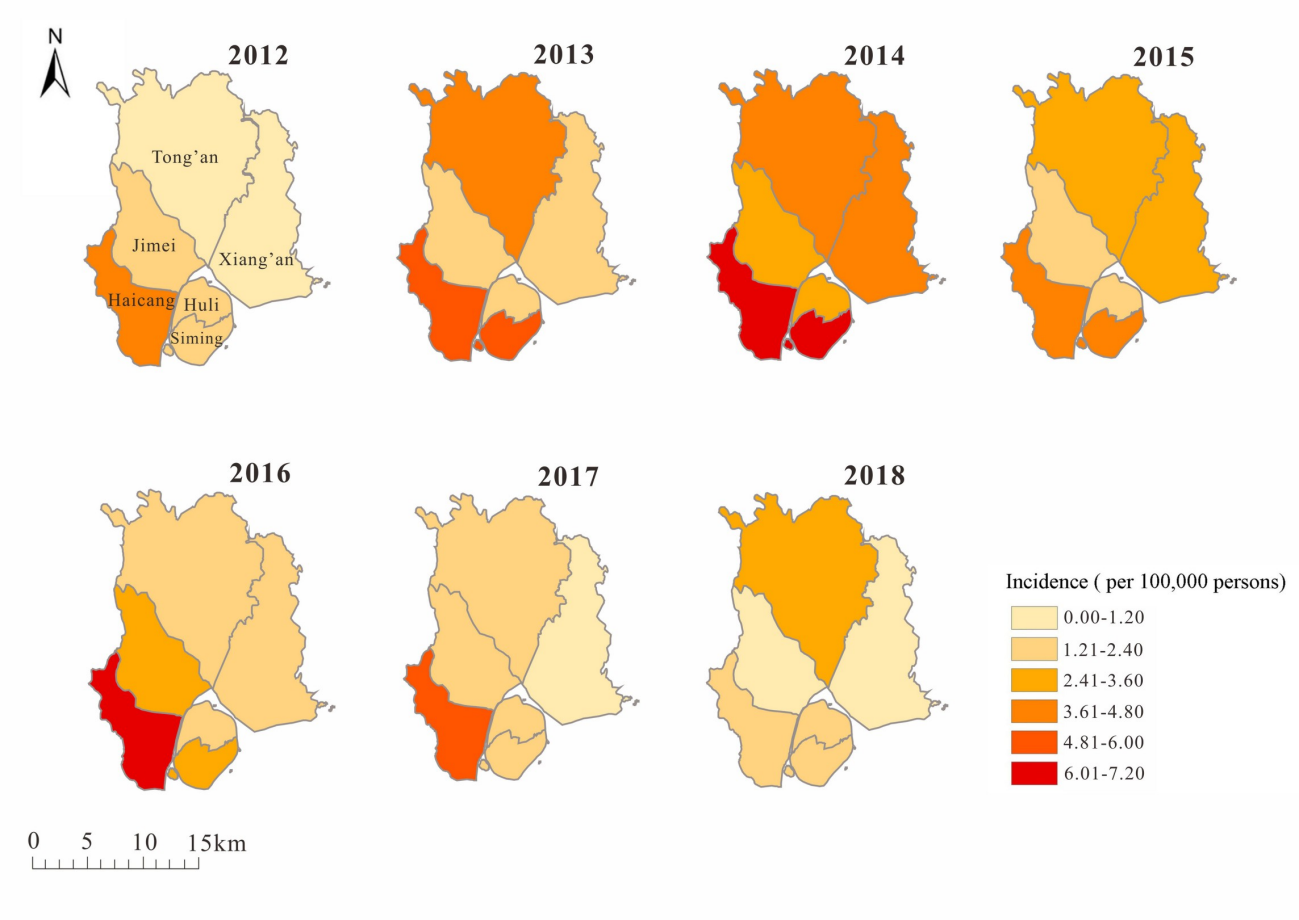
Geographical distribution of TD cases, Xiamen city, China, 2006–2018.

Our results also showed that since 2012, the incidence of TD increased gradually in all the districts and reached a peak in 2014. The incidence in south Xiamen was higher than that in north, with Haicang District having the highest incidence, at 4.75 per 100,000 persons from 2012 to 2018, and Xiang'an District had the lowest incidence, at 1.36 per 100,000 persons from 2006 to 2018 (Fig 5).

**Fig 6:**
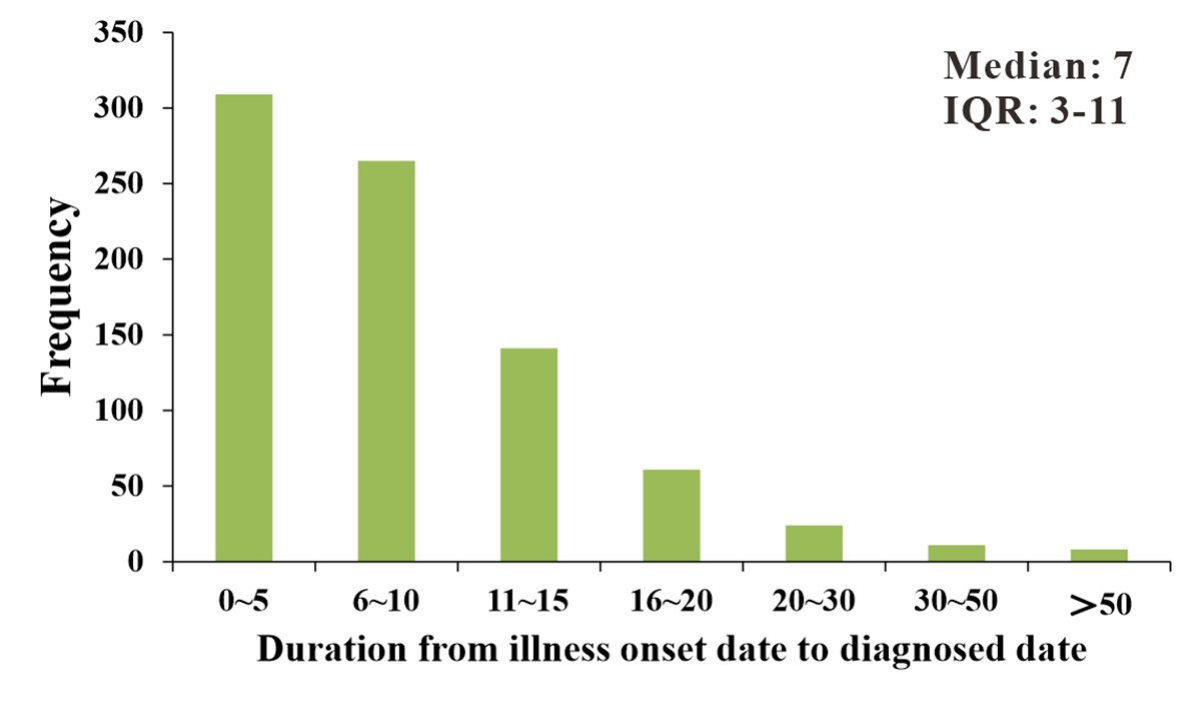
Distribution of duration from illness onset date to diagnosed date of 819 TD cases, Xiamen city, China, 2006–2018.

The median duration from the illness onset date to the diagnosed date (DID) in all cases was 7 days (inter–quartile range [IQR]: 3–11 days) (Fig 6). The DID was shorter than 10 days in 70.09% of cases, shorter than 5 days in 37.73% of cases, and shorter than 2 days in 19.90% of cases (Table 1).

### Host monitoring

**Fig 7:**
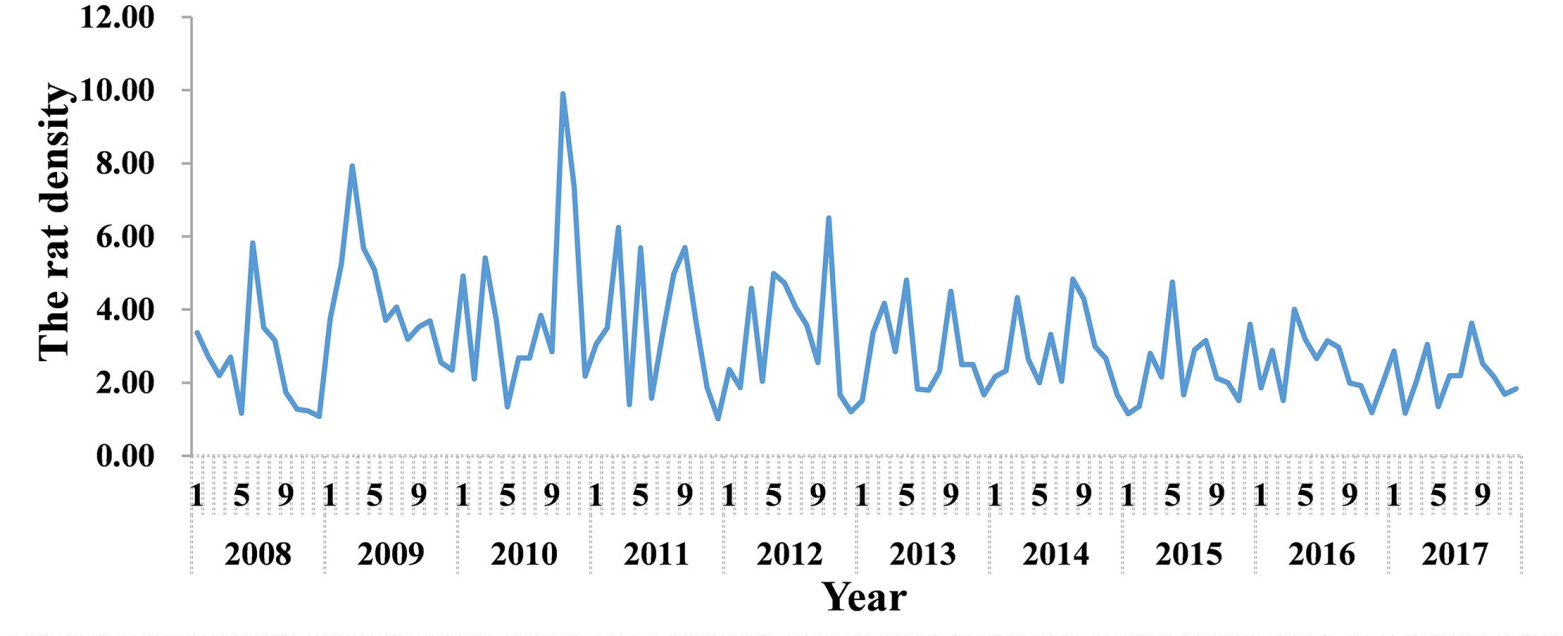
Month distribution of rat density, Xiamen city, China, 2008–2017.

The monitoring results showed that the main hosts were rats (*Rattus norvegicus* and *Rattus flavipectus*), with *R*. *norvegicus* accounting for the largest proportion of rats (73.00%, 887/1215). *Apodemus agrarius* and *Rattus rattoides* were not detected (S2 Table). Additionally, the rat density was highest in March and October every year (Fig 7).

### Results of genome typing of *O*. *tsutsugamushi*

From 2014 to 2015, a total of 100 whole blood samples from suspected patients from clinics and hospitals in Xiamen city, were analyzed. Among them, 24 patients were positive with *O*. *tsutsugamushi*, with a positive rate of 24.00% (24/100). About 168 mouse livers were analyzed, and 53 were positive with *O*. *tsutsugamushi*, with a positive rate of 31.55% (53/168). Of the 77 positive samples, 66 were Karp positive, accounting for 85.71% (66/77), 10 were Gilliam positive, accounting for 12.99% (10/77) and 1 was Kato positive, accounting for 1.30% (1/77). Among the 24 positive human samples, with *O*. *tsutsugamushi* genotypes, 14 were Karp positive, accounting for 25.33% (14/24), 9 were Gilliam positive, accounting for 37.50% (9/24) and 1 was Kato positive, accounting for 4.17% (9/24). Among the 53 positive genotypes from mouse livers, 52 were Karp positive, accounting for 97.11% (52/53) and 1 was Gilliam positive, accounting for 1.89% (1/53).

**Fig 8:**
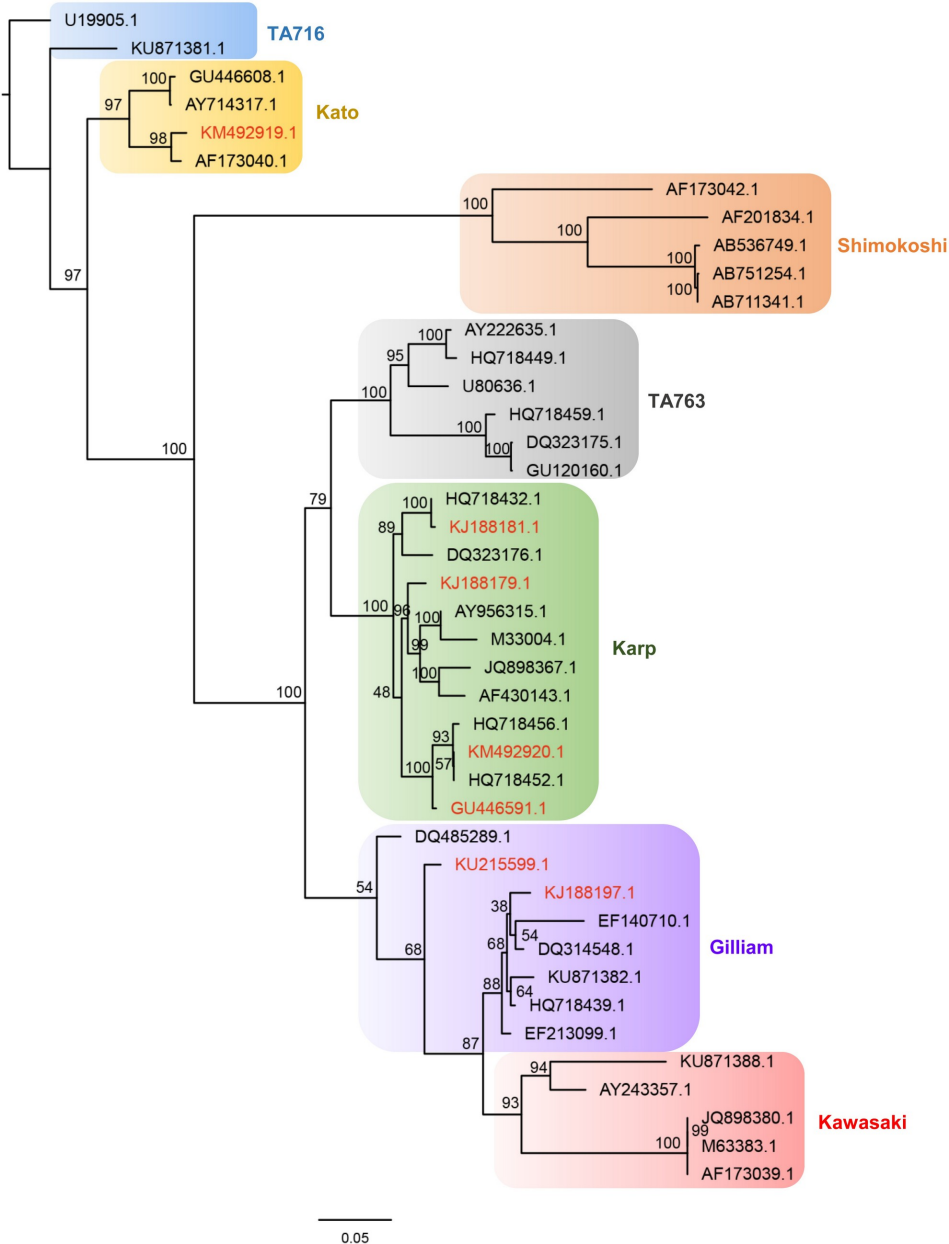
Phylogenetic analysis of *O*. *tsutsugamushi* isolated from host and human TD cases (noted as red color), Xiamen city, China.

The phylogenetic tree of *O*. *tsutsugamushi* isolated from selected host and human TD cases was shown in Fig 8. The phylogenetic analysis showed that out of the seven sequences of hosts and human TD cases obtained in current study from Xiamen, one sequence (KM492919.1) demonstrated the highest similarity to Kato strain with high support. While four sequences (KJ188181.1, KJ188179.1, KM492920.1and GU446591.1) were clustered in Karp strain, and two remaining sequences (KU215599.1 and KJ188197.1) were most closely related to Gilliam strain.

### The relationship between meteorological factors and the reported incidence of TD

**Fig 9:**
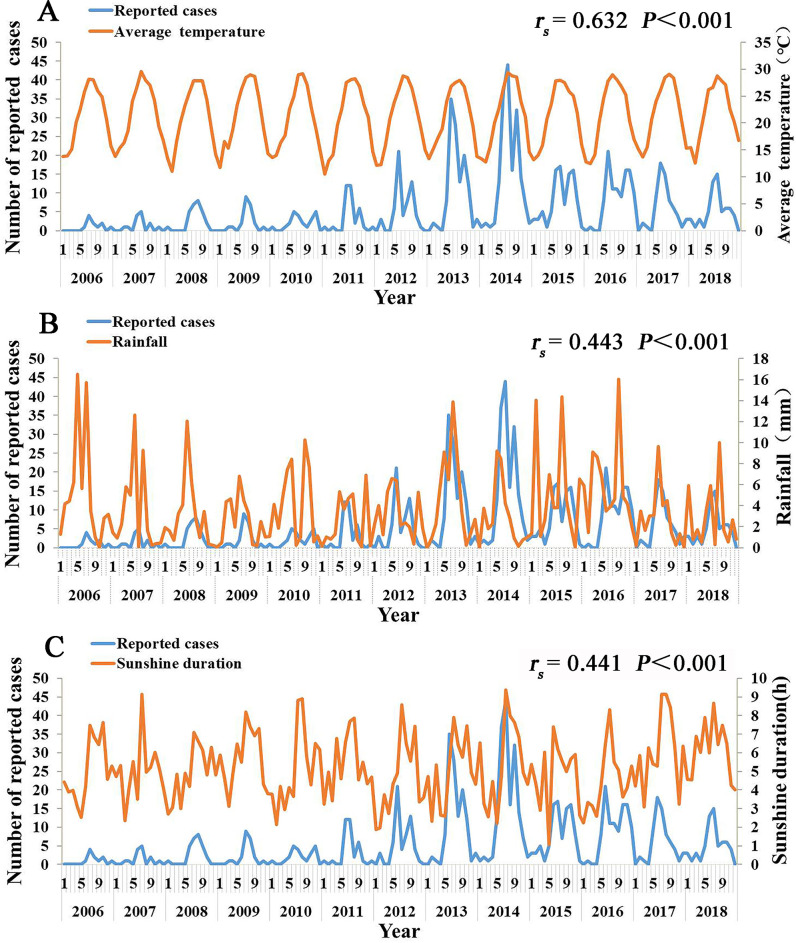
The relationship between meteorological factors and the reported incidence of TD cases, Xiamen city, China, 2006–2018. A, the relationship between monthly average temperature and the reported incidence of TD cases; B, the relationship between rainfall and the reported incidence of TD cases; C, the relationship between monthly average daily sunshine duration and the reported incidence of TD cases.

The results showed that the peak of the number of cases was basically consistent with the peak of average temperature, sunshine duration, and rainfall. The average temperature and sunshine duration were the highest in July. The average temperature reached 28°C and the sunshine duration reached 8 h/day in July. Rainfall was mainly prevalent during summer and autumn, with the highest rainfall reaching 7.18 mm in June. The correlation analysis results showed that there were significant positive correlations between the TD cases and meteorological factors, such as temperature, sunshine duration, and rainfall, with a Spearman’s correlation coefficient (*r*) of 0.620, 0.405, and 0.211, respectively (all *P* < 0.05). Most of the TD cases were associated with higher temperature, more sunshine hours, and more rainfall (Fig 9).

### The relationship between meteorological factors and the rat density

**Fig 10:**
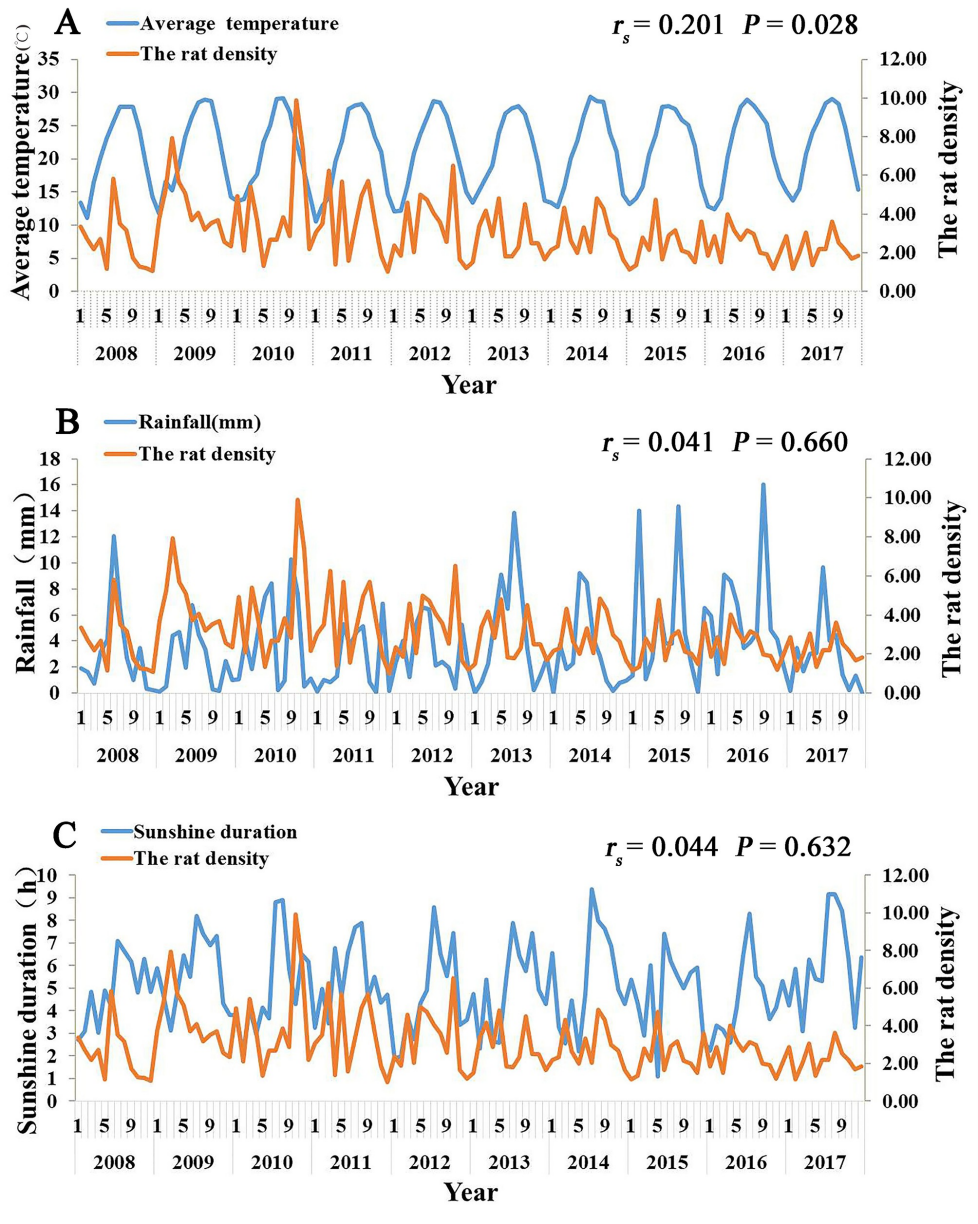
The relationship between meteorological factors and the rat density, Xiamen city, China, 2008–2017. A, the relationship between monthly average temperature and the rat density; B, the relationship between rainfall and the rat density; C, the relationship between monthly average daily sunshine duration and rat density.

As shown in Fig 10, the rat density was basically consistent with the peak of the average temperature. The correlation analysis results showed that there was a significant positive correlation between rat density and average temperature (Spearman’s *r* = 0.201, *P* < 0.05), indicating that the higher temperature was associated with rat density. There were no significant correlation between rat density and rainfall and sunshine duration.

### Diversity and similarity analysis

The results of the occupational characteristics of TD for each case in each district showed that Siming District had the highest *N*, *D*, *H*, and *E* index. Xiang’an District had the lowest *N*, *D*, *H*, and *E* index but had the highest *d* index. Haicang District had the lowest *d* index **(**Table 2**)**. Siming District and Huli District, which are the only 2 bordering islands, had the highest similarity of TD occupational classification. Xiang’an District and Haicang District had low similarity, but the bordering districts Xiang’an District, Jimei District, and Tong’an District showed the lowest similarities (Table 3).

**Table 2:** The constitution of TD by occupational classification in each district, Xiamen city, China, 2006–2018.

District	*N*	Diversity index		*d*	*E*
		*D*	*H*		
Siming	13	0.877	1.632	0.224	0.636
Huli	13	0.874	1.039	0.219	0.405
Jimei	13	0.869	1.305	0.202	0.509
Xiang’an	9	0.734	0.740	0.441	0.289
Tong’an	12	0.877	1.309	0.186	0.527
Haicang	12	0.877	1.407	0.161	0.566

**Table 3:** The similarity of TD according to occupational classification in all districts, Xiamen city, China, 2006–2018.

District	Siming	Huli	Jimei	Tong’an	Xiang’an	Haicang
Siming	1					
Huli	0.944	1				
Jimei	0.899	0.924	1			
Tong’an	0.909	0.927	0.851	1		
Xiang’an	0.822	0.789	0.694	0.756	1	
Haicang	0.918	0.930	0.976	0.857	0.696	1

The index *N*, *D*, *H*, and *d* of the hosts were similar from 2012 to 2017. The year 2018 had different values for the 4 indices, which might be owing to the missing data in June, August, October, and December. However, the years 2014, 2016, and 2017 had much higher values of *E* index (Table 4). A high similarity value of the host characteristics was observed between any 2 years (Table 5).

**Table 4:** The constitution of hosts in each year, Xiamen city, China, 2012–2018.

Year	*N*	Diversity index		*d*	*E*
		*D*	*H*		
2012	4	0.387	0.683	0.755	0.492
2013	3	0.383	0.620	0.749	0.564
2014	2	0.389	0.577	0.736	0.833
2015	3	0.382	0.565	0.746	0.514
2016	2	0.388	0.576	0.737	0.831
2017	2	0.389	0.577	0.736	0.833
2018*	3	0.540	0.690	0.567	0.628

*Note: there is no data in June, August, October, and December, 2018

**Table 5:** The similarity of host in each year, Xiamen city, China, 2012–2018.

Year	2012	2013	2014	2015	2016	2017	2018
2012	1.000						
2013	0.999	1.000					
2014	0.995	0.998	1.000				
2015	0.997	1.000	0.998	1.000			
2016	0.996	0.999	0.999	1.000	1.000		
2017	0.995	0.999	0.999	1.000	1.000	1.000	
2018*	0.941	0.951	0.968	0.954	0.959	0.959	1.000

*Note: there is no data in June, August, October, and December, 2018

## Discussion

TD is one of the most seriously neglected infectious diseases in some countries, including Japan, Taiwan, the Philippines, Indonesia, and Sri Lanka [24,25]. To our knowledge, this is the first study in Xiamen city that used TD cases and meteorological monitoring data for providing useful information on the prevention of TD. This study provides evidence that could help with the prevention and control of TD in Xiamen city, Fujian province, China. The results showed that TD epidemics occurred in the summer and autumn during the year. Therefore, comprehensive public health interventions, including public health education (promotion of good hygiene practices, correct and effective individual protection), deratization and surveillance, should be implemented in Xiamen, especially in areas outside Xiamen Island. We also found that about half of the cases were diagnosed in 7 days from the onset of their illness. This also meant that about 50% of the cases were diagnosed after 7 days from the onset of illness, with only about 19.90% of the cases were diagnosed within 2 days. Therefore, the ability of the surveillance system for diagnosing the disease should be improved for an early treatment. The results also showed that the reported TD incidence in Xiamen city gradually increased since 2006, reached its peak in 2014, and then gradually decreased afterward; the gradual increase may be related to the improved surveillance and diagnosis strategies for TD, and the decrease may be related to the corresponding effective preventive measures. We also observed that the incidence of TD presented an obvious seasonal pattern. The epidemic periods were mainly concentrated in the summer and autumn, with the peak in July. This may be due to the high rainfall in summer and autumn, which makes it easier for chigger mites and rodents to breed.

A study in Taiwan showed that the incidence of scrub typhus was higher in secondary vegetation and intact forests, where chiggers and small mammal hosts are more abundant [26]. During the study period 2006–2018, we found that TD was prominent in farmers, which is consistent with the findings of other study in Asia [27]. The reason might be because the farmers are working in the farmland and hence are more likely to be exposed to the infected chigger mites. Epidemiological studies also found that the number of patients in the age group of 40–60 years was the highest, which could be explained by the fact that people in this age group are more likely to be engaged in agricultural activities and hence had a larger probability of TD infection. Spatial analysis showed that the incidence in areas outside Xiamen island with wider rural coverage, such as Haicang District and Tong’an District were higher than those Siming District or Huli District of Xiamen island. The result is similar to the patterns of geographic distribution in Southeast Asia [8,28,29]. This spatial variation in incidence may be reflected by regional differences in the level of disease diagnosis, reporting and treatment within and outside Xiamen island.

The results of this study showed that the incidence of the TD epidemic periods were in the summer and autumn in Xiamen city. However, the results were inconsistent with studies in other areas or countries. For example, the seasonal distribution in Korea showed that the incidence of TD in autumn was higher than that in Japan, while the incidence of outbreak in other seasons were much higher in Japan [30]. Even in China, there are significant seasonal differences between northern and southern parts of the country. A study in Shandong province [31] found that the period from September to November is the peak season for TD in northern Shandong. The seasonal distribution varies from region to region indicating that the meteorological factors may directly affect the spread of the disease. Therefore, exploring the relationship between the meteorological factors and disease onset will help in the prediction of the epidemic trend of TD disease and preparation for disease control and prevention.

Temperature can affect the occurrence of TD by inhibiting or promoting the activity of chigger mites. The results of the current study suggested that the peak of the number of cases was consistent with the peak of the average temperature, with a significant positive correlation between the cases and temperature, which is similar to the results of Kasuya et al [32]. In Xiamen, the main host animals were *R*. *norvegicus* and *R*. *flavipectus*, and the main vector was *Leptotrombidium deliense*. *Leptotrombidium deliense* was more active in summer, and as we have previously reported, the peak of TD incidence in Xiamen was in July. Thus, temperature may affect human susceptibility to TD by affecting the frequency of mites activity. The positive correlation between the temperature and TD means that global warming may increase the incidence of TD or prolong the epidemic periods of TD, which is a public health concern. However, some reports, such as those in India [33] and Korea [32], showed a negative relationship between the temperature and incidence of scrub typhus. Therefore, our findings may only apply to the areas with the similar climate of Xiamen city, and further research is needed for understanding the effect of temperature on the TD incidence.

We also found that the peak of the number of TD cases was basically consistent with the peak of rainfall. This could be explained by the fact that chigger mites are more likely to grow and attach to rodents in humid environments, further affecting the onset of TD. A study in Chile revealed that chigger mites survive and reproduce well at a relative humidity of above 50% but their activity reduce when the relative humidity is below 50% [34]. Around the end of 2014, there was a dissociation in the general consistent trend between rainfall and reported cases, with the number of cases remaining high even with a drop in the rainfall level, which may be because the reported cases peaked in 2014 but the rainfall level fluctuated with the season every year. We found that the number of cases were positively correlated with the sunshine duration, which may be owing to the fact that the longer duration of sunshine is associated with longer time for outdoor activities, which contact with the vegetation environment, and hence, people are more likely to be exposed to infected mites, leading to the increase of scrub typhus infection [8].

Rodents are an important intermediate host for the transmission of *O*. *tsutsugamushi*. Moreover, owing to different rodent species, the infection intensity and feeding success rate of chigger mites vary greatly [35,36]. During the 6 years of monitoring from 2012 to 2018, we found that the main hosts were *R*. *norvegicus and R*. *flavipectuss*, among which *R*. *norvegicus* accounted for the largest proportion. Therefore, special attention should be paid to *R*. *norvegicus* and *R*. *flavipectus* when rodent control is considered as a feasible strategy for reducing the TD burden. The results of the correlation analysis showed that the rodent density positively correlated with the average temperature, indicating that the average temperature could directly or indirectly favor rodents’ survival and reproduction, thereby, increasing rodent density. In addition, Spearman’s correlation coefficient between the reported cases and average temperature is the largest, which indicates that higher temperature is associated with higher rodent density, increased chigger mites, and increased incidence of TD. The specific mechanism, however, needs to be further explored. Furthermore, the phylogenetic analysis showed that there were 3 strains of *O*. *tsutsugamushi* in Xiamen; therefore, the surveillance should be strengthened, and further analysis should be conducted for finding any differences in the epidemic characteristics, so that appropriate prevention and control measures could be implemented.

There are still some limitations in our study. First, data on the cases were collected from a passively reported system, so other cases might not have been monitored or recorded. Second, this study is an ecological study. When we studied the effects of meteorological factors on TD incidence and rodent density, other confounding factors could not be excluded, which may cause confounding bias. In addition, we did not include monitoring data on chigger mites. Thus, data on chigger mites distribution in Xiamen should be included in future studies. Finally, genome typing was only performed between 2014 and 2015, and the genome of *O*. *tsutsugamushi* could be different or might have changed in other years.

In conclusion, this study showed that meteorological factors were associated with the incidence of TD and the host of *O*. *tsutsugamushi* in Xiamen city, China. The incidence of TD was seasonal, and the epidemic period was mainly from May to October. The highest rat density was observed in March and October every year. Moreover, average temperature, duration of sunshine, and rainfall were positively associated with TD incidence. Average temperature was also positively associated with rat density. These findings reveal that the activity of the host of *O*. *tsutsugamushi* was only be affected by temperature. However, TD incidence was not only affected by temperature but also by the duration of sunshine and rainfall.

## Supporting information

S1 Fig Administrative divisions, Xiamen city, Fujian Province, China. (TIF)

S1 Table The PCR detection sequence and primers for *O*. *tsutsugamushi*. (XLSX)

S2 Table Number of various kinds of rat, Xiamen city, China, 2012–2018. (XLSX)
