# Supplementary figures and images for: Epidemiology of tsutsugamushi disease and its relationship with meteorological factors in Xiamen city, China
Source: PLoS Negl Trop Dis. 2020 Oct 15;14(10):e0008772. doi: 10.1371/journal.pntd.0008772 (PMC7591240; doi:10.1371/journal.pntd.0008772)

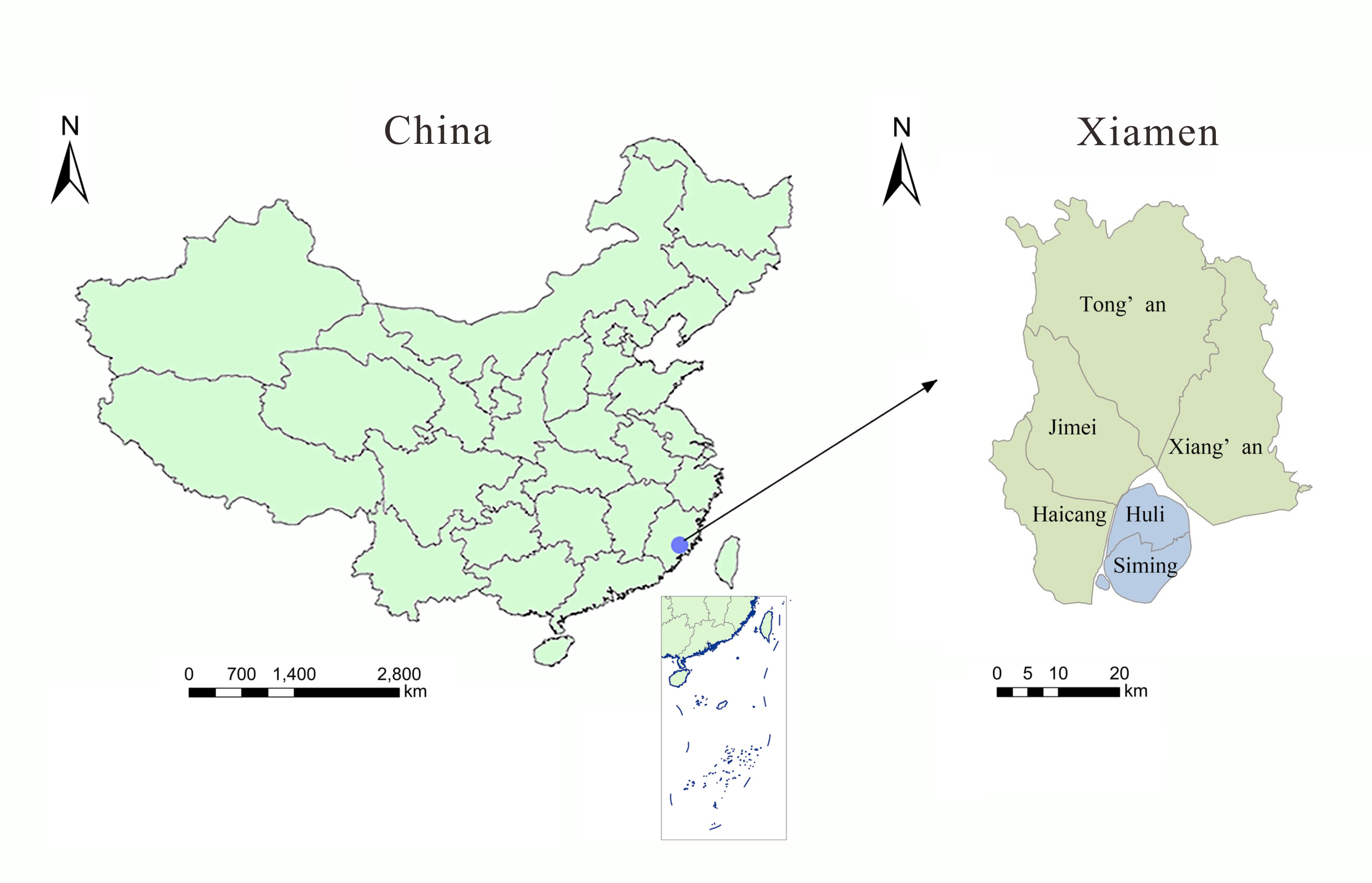

Supplement: S1 Fig — (TIF) [file pntd.0008772.s001.tif]
